# Computed tomography angiography-based radiomics model for predicting carotid atherosclerotic plaque vulnerability

**DOI:** 10.3389/fneur.2023.1151326

**Published:** 2023-06-16

**Authors:** Dezhi Shan, Siyu Wang, Junjie Wang, Jun Lu, Junhong Ren, Juan Chen, Daming Wang, Peng Qi

**Affiliations:** ^1^Department of Neurosurgery, Beijing Hospital, National Center of Gerontology, Institute of Geriatric Medicine, Chinese Academy of Medical Sciences, Beijing, China; ^2^Graduate School of Peking Union Medical College, Beijing, China; ^3^Department of Ultrasound, Beijing Hospital, National Center of Gerontology, Institute of Geriatric Medicine, Chinese Academy of Medical Sciences, Beijing, China; ^4^Department of Radiology, Beijing Hospital, National Center of Gerontology, Institute of Geriatric Medicine, Chinese Academy of Medical Sciences, Beijing, China

**Keywords:** CTA radiomics, carotid atherosclerotic plaque, vulnerability, CEUS, machine learning algorithms

## Abstract

Vulnerable carotid atherosclerotic plaque (CAP) significantly contributes to ischemic stroke. Neovascularization within plaques is an emerging biomarker linked to plaque vulnerability that can be detected using contrast-enhanced ultrasound (CEUS). Computed tomography angiography (CTA) is a common method used in clinical cerebrovascular assessments that can be employed to evaluate the vulnerability of CAPs. Radiomics is a technique that automatically extracts radiomic features from images. This study aimed to identify radiomic features associated with the neovascularization of CAP and construct a prediction model for CAP vulnerability based on radiomic features. CTA data and clinical data of patients with CAPs who underwent CTA and CEUS between January 2018 and December 2021 in Beijing Hospital were retrospectively collected. The data were divided into a training cohort and a testing cohort using a 7:3 split. According to the examination of CEUS, CAPs were dichotomized into vulnerable and stable groups. 3D Slicer software was used to delineate the region of interest in CTA images, and the Pyradiomics package was used to extract radiomic features in Python. Machine learning algorithms containing logistic regression (LR), support vector machine (SVM), random forest (RF), light gradient boosting machine (LGBM), adaptive boosting (AdaBoost), extreme gradient boosting (XGBoost), and multi-layer perception (MLP) were used to construct the models. The confusion matrix, receiver operating characteristic (ROC) curve, accuracy, precision, recall, and f-1 score were used to evaluate the performance of the models. A total of 74 patients with 110 CAPs were included. In all, 1,316 radiomic features were extracted, and 10 radiomic features were selected for machine-learning model construction. After evaluating several models on the testing cohorts, it was discovered that model_RF outperformed the others, achieving an AUC value of 0.93 (95% CI: 0.88–0.99). The accuracy, precision, recall, and f-1 score of model_RF in the testing cohort were 0.85, 0.87, 0.85, and 0.85, respectively. Radiomic features associated with the neovascularization of CAP were obtained. Our study highlights the potential of radiomics-based models for improving the accuracy and efficiency of diagnosing vulnerable CAP. In particular, the model_RF, utilizing radiomic features extracted from CTA, provides a noninvasive and efficient method for accurately predicting the vulnerability status of CAP. This model shows great potential for offering clinical guidance for early detection and improving patient outcomes.

## Introduction

Ischemic stroke (IS) is associated with high morbidity and mortality, resulting in a high socioeconomic burden (1, 2, 48). Carotid atherosclerotic plaque (CAP) is closely related to the occurrence of IS (3, 4). Vulnerable plaques are prone to rupture and hemorrhage under the action of various hemodynamics, leading to the occurrence of clinical symptoms, which is an important mechanism leading to IS (5, 6). Therefore, it is of great clinical importance to apply assessment and early clinical intervention to prevent cerebrovascular events in patients with vulnerable plaques. Pathological studies suggest that neovascularization is directly related to plaque vulnerability (7), which is also consistent with the findings from clinical studies (8, 9).

Neovascularization could promote plaque inflammatory response and accelerate foam cell aggregation (10, 11), producing a larger lipid necrotic core and increased fibrinolysis, thus resulting in a thinner fibrous cap and aggravating plaque vulnerability. Neovascularization in plaques is considered an emerging biomarker related to plaque vulnerability (12, 13). Contrast-enhanced ultrasound (CEUS) could detect neovascularization in CAPs and assess the vulnerability of CAPs (14). The status of plaque enhancement in carotid atherosclerosis under CEUS was associated with the vulnerability of CAPs (15, 16).

Computed tomography angiography (CTA) is a widely used clinical cerebrovascular examination (17). CTA has a high resolution, and the morphology of the lumen and plaque can be accurately judged. Both pathological and clinical studies suggest that plaque morphology may reflect plaque vulnerability (18–20). Therefore, it could be speculated that the lumen or plaque morphology reflected by CTA is highly correlated with plaque vulnerability.

However, the relationship between existing morphological features obtained by CTA and the vulnerability status of CAPs was not intuitive; therefore, more efforts are needed to get more intuitive and meaningful morphological features.

“Artificial intelligence (AI) technology” is an emerging technical science to simulate and expand human intelligence (21, 22). As a part of AI, machine learning (ML) has been widely used in many medical fields, especially for disease prediction and diagnosis (23–25). Moreover, radiomics is a medical imaging field involving the extraction and analysis of quantitative features from medical images. Compared with traditional imaging phenotypic features, more objective and quantitative imaging features that are difficult to identify with the naked eye could be obtained using radiomics techniques (26–28). The model established by radiomic features with ML algorithms showed great predictive performance (29, 30). Owing to the low cost of clinical application, it could easily provide individual diagnosis and treatment services. However, few ML diagnostic models for predicting plaque vulnerability based on radiomic features of CTA have been reported. This study aimed to identify the radiomic features associated with the neovascularization of CAP and to construct a prediction model based on CTA radiomic features, which may guide the detection of vulnerable carotid plaque and treatment decisions.

## Materials and methods

### Study population

The included patients were treated for atherosclerosis stenosis of the carotid artery at Beijing Hospital from January 2018 to December 2021. The inclusion criteria for this study were (1) adult patients over 18 years old, (2) a diagnosis of CAP on CTA and CEUS, and (3) relevant CTA and CEUS examinations that were performed simultaneously, not exceeding 3 weeks. The exclusion criteria included (1) cases without available clinical records and (2) CTA images of poor quality that could not extract radiomic features.

### Clinical and imaging data

Clinical and CTA data were collected retrospectively. The clinical information included the patient's sex, age, smoking history, alcohol history, and history of hypertension and diabetes mellitus. Plaques were divided into two groups based on the status of plaque enhancement in CEUS as follows: the vulnerable and stable plaque groups. The contrast agent development of plaque was utilized as an indicator to evaluate angiogenesis, and a plaque was considered stable if no contrast agent development was observed, indicating the absence of new blood vessels.

Conversely, a vulnerable plaque was identified if a single or simultaneous development of contrast agent was observed at the bottom, top, and shoulder of the plaque, indicating the presence of new blood vessels. Carotid CTA scans were performed with a 320 × 0.5-mm detector row CT scanner (AquilionONE, Canon Medical Systems). Scanning parameters were as follows: 80 kV, 100 mAs, a cover range of 16 cm, reconstruction with adaptive iterative dose reduction, and a layer thickness of 0.5 mm.

### Image segmentation and feature extraction

3D Slicer software was used to delineate ROI on the obtained CTA images. In the image segmentation process, the threshold segmentation method was used to delineate the area with a fixed threshold range, and then, the selected area was adjusted manually. The included segment was the carotid artery corresponding to the CAP. Two general radiologists with 5 and 7 years of experience in head CTA independently completed the work using 3Dslicer 4.10.1. The segmentation was performed using 3D segmentation, and a strictly consistent criterion was followed to modify the segmentation and avoid calcification. The Pyradiomics package in Python software was used to perform radiomic feature extraction, and from all features, three types of features were mainly extracted: (1) first-order features, mainly included features such as energy, entropy, kurtosis, and skewness; (2) shape features, which mainly included features such as volume, surface, sphericity, compactness, diameter, and flatness; (3) texture features, usually based on different matrices to extract texture features, such as Gray Level Co-occurrence Matrix (GLCM) features, Gray Level Size Zone Matrix (GLSZM) features, Gray Level Run Length Matrix (GLRLM) features, Neighboring Gray Tone Difference Matrix (NGTDM) features, and Gray Level Dependence Matrix (GLDM) features. Shape features were extracted from the original image, while first-order features and texture features were extracted from both the original image and the original image transformed by filters, including Laplacian of Gaussian (LoG), wavelet decompositions with all possible combinations of high-(H) or low-(L) pass filters in each of the three dimensions (HHH, HHL, HLH, LHH, LLL, LLH, LHL, HLL), and exponential and gradient filters.

### Radiomic feature selection

The intraclass correlation coefficient (ICC) was calculated on a subset of 50 images to evaluate the consistency and reliability of the radiomic features obtained from the segmented images, and features with an ICC of >0.9 were selected for further study. The independent samples *t*-test was used to identify significantly different variables between the vulnerable and stable plaque groups. Features with a *P* < 0.05 were considered statistically significant. Radiomic features that met the requirements for being different between groups were considered. The basic radiomic features of vulnerable plaques were identified as the most highly expressed radiomic features in the vulnerable plaque group. Furthermore, the radiomic features of group differences were selected for subsequent model construction.

### Predictive model construction and evaluation

**Figure 1:**
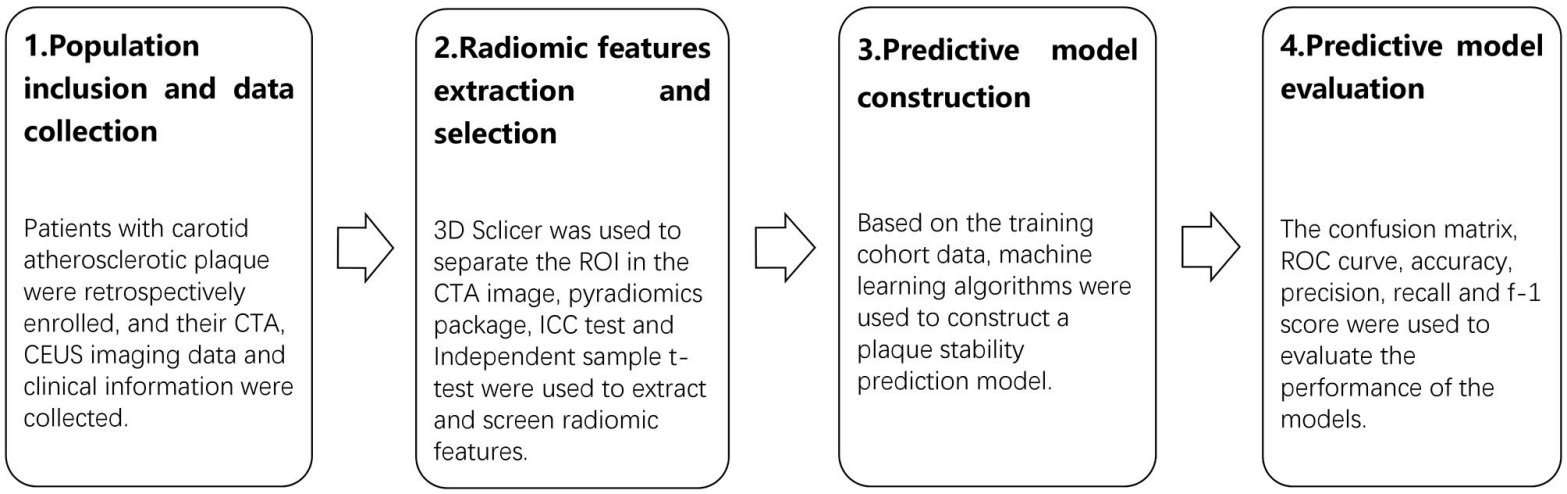
The technical roadmap of the research. The study consisted of four stages: population inclusion and data collection, radiomic features extraction and selection, predictive model construction, and predictive model evaluation.

Machine learning algorithms were used for model building, which included logistic regression (LR), the support vector machine (SVM), the random forest (RF), the light gradient boosting machine (LGBM), adaptive boosting (AdaBoost), extreme gradient boosting (XGBoost), and multi-layer perception (MLP) based on the ML frameworks Scikit-learn (31) and XGBoost in Python (32). The data were divided into training and testing cohorts at a ratio of 7:3. A prediction model was constructed based on the radiomic features related to CAP vulnerability. Model fitting was performed with the training cohort data to construct a plaque vulnerability prediction model. Each of the seven models was constructed using training cohort data. Confusion matrices were constructed for the training and testing cohorts of each model. The corresponding receiver operating characteristic (ROC) curve was generated, and the predictive performance of the models was evaluated by the area under the curve (AUC). The t-distribution method was used to calculate the 95% confidence interval (CI) of the AUC value. The accuracy, precision, recall, and f-1 score evaluation metrics were calculated to evaluate the model's effectiveness. In addition, a predictive model based on the RF algorithm was built using clinical information from the patients included in the study. The model with the best performance was selected for the plaque vulnerability prediction model. The technical roadmap is shown in Figure 1.

### Statistical analysis

Statistical analyses were performed using R software. The ROC curves were generated to assess the performance of the radiomics model in the training and testing cohorts. The accuracy, precision, recall, and f-1 score were used to measure the comprehensive level of the model. The significance level was set at a *p*-value of = 0.05 for the basic statistical analyses.

## Results

### Clinical features

**Figure 2:**
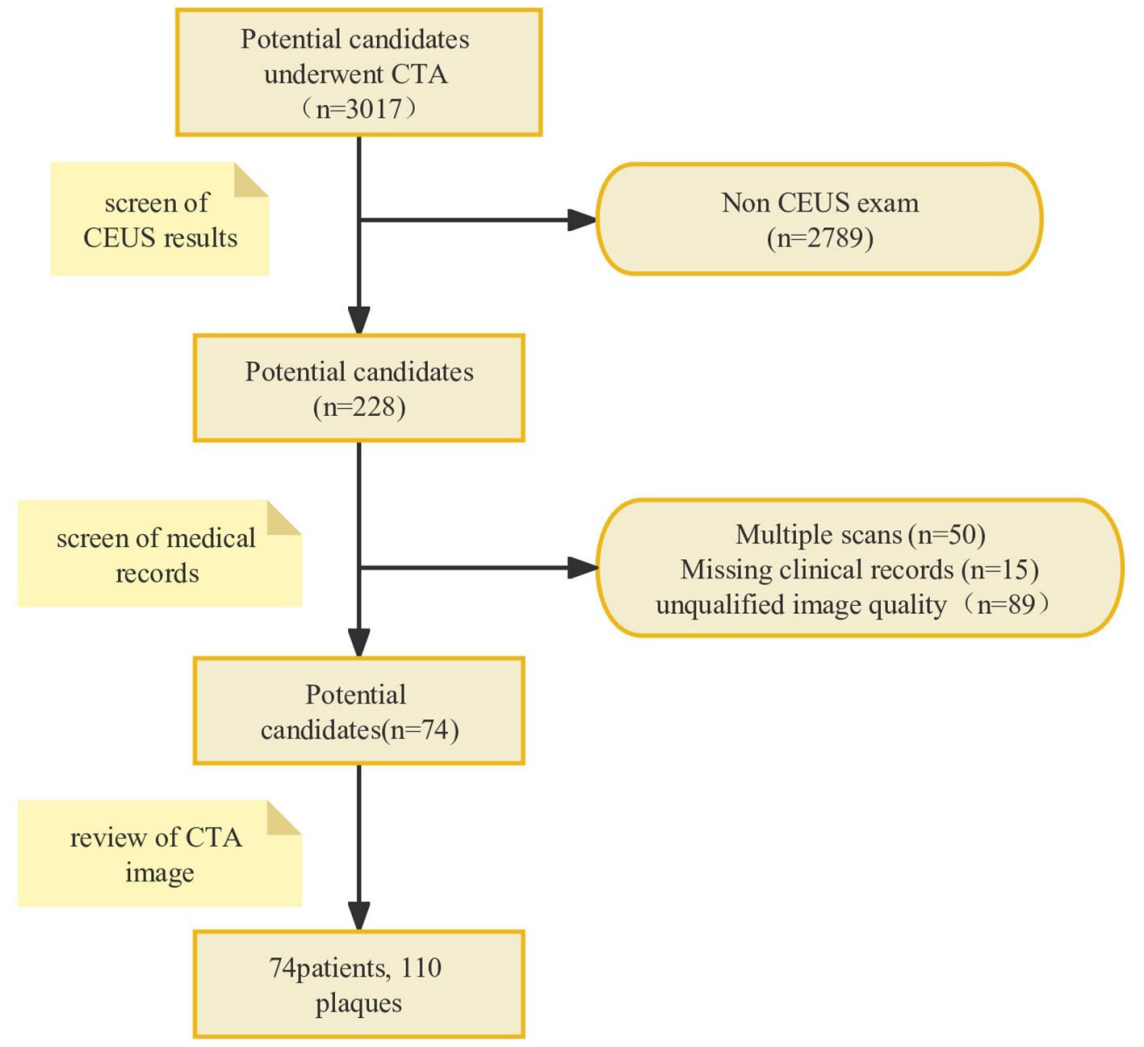
The flowchart of the patient inclusion process. A total of 74 patients with 110 CAPs were included after analyzing patient imaging data and medical records according to the inclusion and exclusion criteria. CAP, carotid atherosclerotic plaque.

A total of 74 patients (mean age, 66.9 ± 8.82 years; 85.1% men) with 110 CAPs were included. A flowchart was drawn to describe the patient inclusion process (Figure 2). The baseline characteristics of the patients are shown in Table 1. Out of a total of 74 patients, 30 were identified during routine physical check-ups, while the remaining 44 were detected during post-stroke examinations. Patients with stroke accounted for 59.5% of the total number of patients. Among the stroke patients, a total of 64 plaques were discovered, out of which 40 were linked to ipsilateral strokes. Additionally, the median value for enrolled patients with carotid artery stenosis was 64%. After being identified through CEUS examination, it was found that there were 50 stable plaques and 60 vulnerable plaques among a total of 110 plaques in the examined area.

**Table 1:** Study population characteristics.

**Characteristics**	**Patients (*N* = 74)**
Age, mean ± SD, yr	66.9 ± 8.82
Men, *n* (%)	63(85.1%)
Smoking history, *n* (%)	41(55.4%)
Alcohol history, *n* (%)	28(37.8%)
Hypertension, *n* (%)	52(70.3%)
Diabetes mellitus, *n* (%)	29(39.2%)

### Selected stable features

In total, 1,316 radiomic features were extracted, which included 252 first-order features, 14 shape features, and 1,050 texture features. Texture features consisted of 336 GLCM features, 224 GLSZM features, 224 GLRLM features, 70 NGTDM features, and 196 GLDM features. After performing ICC testing, a total of 990 radiomic features that met the required conditions were selected for further analysis (Supplementary Figure S1). Subsequently, 10 radiomic features were identified as significantly different between the vulnerable and stable plaque groups using the *t*-test. These features were chosen for further model-building processes based on their potential to effectively differentiate between the various types of plaques being studied. The selected features included “square_glszm_ZoneEntropy, square_glszm_SizeZoneNonUniformityNormalized, wavelet- LLL_glcm_MaximumProbability, wavelet-LLL_glcm_Joint Energy, original_glcm_JointEnergy, wavelet- HHL_glszm_Low GrayLevelZoneEmphasis, wavelet-HHH_glszm_GrayLevelNon UniformityNormalized, wavelet- LLL_firstorder_Uniformity, wavelet-LLH_glszm_LargeAreaHighGrayLevelEmphasis, and wavelet- LLH_firstorder_Kurtosis.” Based on these differences, high-expressing radiomic features in vulnerable plaques were selected as the basic radiomic features of vulnerable plaques, including “square_glszm_SizeZoneNonUniformityNormalized, wavelet-LLL_glcm_MaximumProbability, wavelet-LLL_glcm_JointEnergy, original_glcm_JointEnergy, wavelet-HHL_glszm_LowGrayLevelZoneEmphasis, wavelet-HHH_glszm_GrayLevelNonUniformityNormalized, and wavelet-LLL_firstorder_Uniformity.”

### Predictive model construction and evaluation

**Figure 3:**
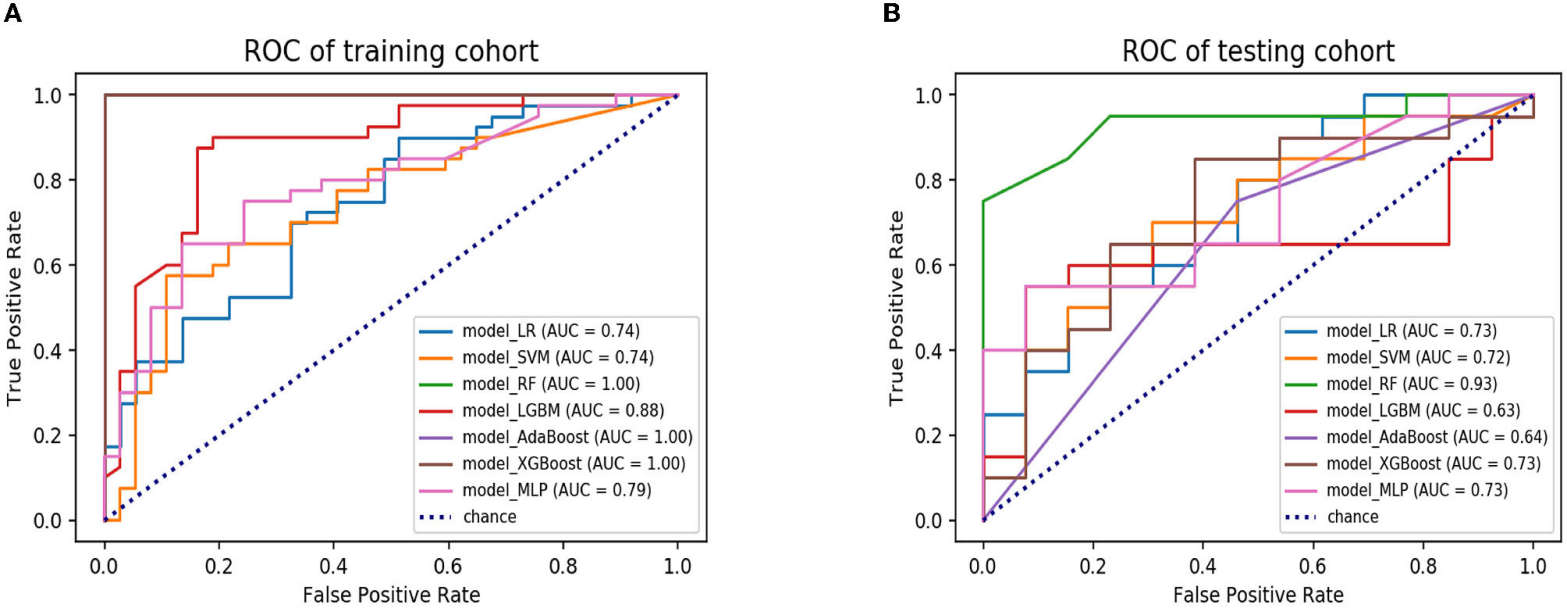
The ROC curves of seven ML models in the training and testing cohorts. **(A)** ROC curves of model_LR, model_SVM, model_RF, model_LGBM, model_AdaBoost, model_ XGBoost and model_MLP in the training cohort. **(B)** ROC curves of model_LR model_ SVM, model_RF, model_LGBM, model_AdaBoost, model_ XGBoost, and model_ MLP in the testing cohort. ROC, receiver operating characteristic; ML, machine learning; LR, logistic regression; SVM, support vector machine; RF, random forest; LGBM, light gradient boosting machine; AdaBoost, adaptive boosting; XGBoost, extreme gradient boosting; MLP, multi-layer perception.

A total of seven ML algorithms were applied to the dataset, resulting in the construction of seven ML models. These models were named as follows: model_LR, model_SVM, model_RF, model_LGBM, model_AdaBoost, model_XGBoost, and model_MLP. The AUC values of the models are shown in Table 2. The confusion matrices of the training and testing cohorts were constructed. The AUC values of model_LR, model_SVM, model_RF, model_LGBM, model_AdaBoost, model_XGBoost, and model_MLP in training cohorts were 0.74, 0.74, 1.00, 0.88, 1.00, 1.00, and 0.79, respectively. Moreover, the AUC values of the testing cohorts were 0.73, 0.72, 0.93, 0.63, 0.64, 0.73, and 0.73. The ROC curves of all models in the testing cohorts are shown in Figure 3. The accuracy, precision, recall, and f-1 score of models are shown in Table 3. After evaluating various combinations of hyperparameters, it was observed that model_RF with n_estimators=35 and max_depth=20 as their main parameters exhibited the most superior performance compared to other configurations. In addition, a model named model_clinical was built using clinical information based on the RF algorithm. The AUC values for the training and testing cohorts were recorded as 0.90 and 0.61, respectively, and the model's accuracy, precision, recall, and f-1 score were measured as 0.55, 0.54, 0.55, and 0.54, respectively. Model_RF outperformed model_clinical significantly, indicating that it is more effective and efficient in accomplishing the task (Supplementary Figure S2).

**Table 2:** AUC of ML models.

**Model**	**AUC (95%CI)**
Model_LR	0.731 (0.651–0.811)
Model_SVM	0.717 (0.713–0.721)
Model_RF	0.933 (0.880–0.985)
Model_LGBM	0.635 (0.581–0.688)
Model_AdaBoost	0.644 (0.471–0.817)
Model_XGBoost	0.727 (0.604–0.849)
Model_MLP	0.729 (0.637–0.820)

95% CIs of AUC value of model_LR, model_SVM, model_RF model_LGBM, model_AdaBoost, model_XGBoost and model_MLP in the testing cohort. AUC, area under the curve; CI, confidence interval; LR, logistic regression; SVM, support vector machine; RF, random forest; LGBM, light gradient boosting machine; AdaBoost, adaptive boosting; XGBoost, extreme gradient boosting; MLP, multi-layer perception.

**Table 3:** Performance of ML models.

**Model**	**Accuracy**	**Precision**	**Recall**	**F1-score**
Model_LR	0.61	0.61	0.61	0.61
Model_SVM	0.64	0.64	0.64	0.64
Model_RF	0.85	0.87	0.85	0.85
Model_LGBM	0.64	0.66	0.64	0.64
Model_AdaBoost	0.67	0.66	0.67	0.66
Model_XGBoost	0.70	0.70	0.70	0.70
Model_MLP	0.58	0.58	0.58	0.58

The accuracy, precision, recall, and f1-score of model_LR, model_SVM, model_RF, model_LGBM, model_AdaBoost, model_XGBoost and model_MLP in the testing cohort.

## Discussion

Carotid atherosclerosis is a common mechanism of IS (33, 34). The vulnerability of CAP is closely related to the occurrence of stroke (35, 36). The rupture of vulnerable CAPs causes thromboembolism, which could lead to IS. Therefore, early identification of vulnerable CAPs is of great significance for improving the prognosis of patients.

CTA has been widely used in the assessment of CAPs based on its non-invasiveness and wide availability of accurate information. Previous studies have shown that the shape of CAPs is closely related to plaque vulnerability. The carotid artery lumen could be detected through CTA examination, and then, the shape characteristics of the plaque in the lumen could be obtained to predict the vulnerability of the plaque. While previous studies mostly focused on traditional plaque features, such as plaque volume, neovascularization, and inflammatory features, which have been extensively identified as biomarkers of carotid plaque vulnerability (12, 37), existing morphological features are not accurate enough. Moreover, radiomics, capable of high-throughput extraction of radiomic features, holds great potential for medical imaging to provide more information for clinical decision-making in a non-invasive manner. Currently, studies have demonstrated promising results in predicting the clinical symptoms of carotid artery atherosclerosis patients by utilizing radiomic features and machine-learning algorithms that are based on patient images. As noted in previous studies, the imaging modalities used for diagnosis primarily consist of magnetic resonance imaging (MRI), ultrasound, and computed tomography angiography (CTA) (38–41). Moreover, recent research has indicated that CT texture analysis (CTTA) may play an important role in identifying vulnerable plaques in patients with carotid artery atherosclerosis. The study indicates that CTTA has the potential to become a novel risk stratification tool for carotid artery atherosclerosis patients, helping to identify patients with a higher risk of stroke and TIA (42). However, the aforementioned studies were mainly grouped based on the symptoms of patients, and there were still few radiomics models to predict the vulnerability of CAPs. In this study, the radiomics approach was used to extract radiomic features from conventional CTA images, and related machine-learning models were constructed to predict the vulnerability of CAPs. Just as mentioned, the morphology of CAPs might reflect vulnerability. Therefore, the blood vessels where the CAPs are located were selected as the ROI to predict the vulnerability of CAPs.

In this retrospective study, various features were extracted using PyRadiomics. The top three radiomic features were square_glszm_ZoneEntropy, square_glszm_SizeZoneNonUniformityNormalized, and wavelet-LLL_glcm_MaximumProbability. Square_glszm_ZoneEntropy refers to the ZoneEntropy of glszm obtained by applying the square filter. A lower value of this feature may indicate the formation of new blood vessels within the plaque, which could suggest increased plaque vulnerability. Square_glszm_SizeZoneNonUniformityNormalized refers to the SizeZoneNonUniformityNormalized (SZNN) of glszm obtained by applying the square filter.

A higher value of this feature indicates greater homogeneity among zone size volumes in the image, which suggests plaque vulnerability. Wavelet-LLL_glcm_MaximumProbability refers to the maximum probability of glcm obtained by applying the wavelet-LLL filter. An increase in this feature's value indicates the presence of neovascularization within the plaque, which is another indicator of plaque vulnerability. Moreover, wavelet-LLL_glcm_MaximumProbability has been reported to distinguish sinonasal primary lymphomas from squamous cell carcinomas (43).

ML has made great progress in disease prediction with the improvement of computing power and the update of algorithms (44–46). Various machine learning models, such as LR, RF, SVM, LGBM, Adaboost, XGBoost, and MLP, have been applied in different scenarios, each with its own strengths. For instance, LR, RF, Adaboost, XGBoost, and MLP are capable of performing classification tasks, while LGBM and SVM can be utilized for regression tasks. Additionally, large-scale datasets benefit from the faster training speed and higher accuracy of LGBM, while XGBoost exhibits better overall performance. However, RF, Adaboost, and XGBoost are ensemble learning methods that leverage multiple decision trees to obtain better results, while SVM, LGBM, and MLP are single models.

Therefore, choosing the appropriate machine learning model for specific data and tasks is crucial. These common models offer a diverse range of choices that can cater to various fields. In summary, these ML models have differences in application scenarios, performance, and efficiency, requiring decisions based on specific problems and data characteristics. The current study constructed seven predictive models to identify the vulnerability of CAP. Among these models, model_RF demonstrated superior performance. Model_RF had better AUC, accuracy, precision, recall, and f-1 scores than the other models. In both the training and testing cohorts, model_RF effectively predicted the vulnerability of CAPs by predicting vulnerable and stable plaques. Model_RF outperformed traditional model_LR in the testing cohort because the emerging ML algorithm has the advantage of processing massive data and many parameters for configuration optimization, making it more flexible than traditional model_LR.

Furthermore, the performance of model_RF was superior to that of model_clinical, which relied solely on clinical features. This indicates that the radiomics-based ML model outperforms traditional clinical features in assessing the stability of CAPs. Furthermore, radiomic feature calculation is a fast and automatic process once the ROI has been delineated; therefore, the selected radiomic signature can be integrated with the automatic 3D carotid segmentation system (47) for a comprehensive CAP detection and vulnerability prediction, which could contribute to clinical decision-making in CAPs.

The study has some limitations. First, the retrospective enrollment utilized in the study was determined by our clinicians, which may introduce some selection bias and limit the generalizability of the findings. Second, this study selected the luminal area where the CAP is located as the ROI because CAP morphology has an excellent predictive value for CAP vulnerability. However, if the characteristics of the CAPs themselves could be combined, it might further improve the prediction ability, which is also our follow-up research direction. Additionally, the sample size in this study was relatively small, which may limit the generalizability of our findings. Although we have attempted to mitigate this limitation by carefully selecting our cohort and applying rigorous statistical methods, future studies with larger and more diverse cohorts will be needed to validate and extend our results. This study obtained radiomic features associated with the neovascularization of CAP. Model_RF with CTA radiomics was constructed to predict the vulnerability status of CAP. The current study serves as an important initial step toward developing more accurate and efficient diagnostic tools for diagnosing and treating CAP. Although our model_RF with CTA radiomics showed high accuracy in predicting the vulnerability status of CAP, further validation in larger cohorts is necessary to confirm its clinical utility. Therefore, prospective studies are needed to further validate its classification ability and assess its potential clinical impact. In general, our study emphasizes the potential of radiomics-based models to enhance the accuracy and efficiency of diagnosing vulnerable CAP. Specifically, the model_RF offers a non-invasive and efficient approach to predicting the vulnerability status of CAP by utilizing radiomic features extracted from CTA. The model may enable earlier detection of vulnerable CAPs, improving patient outcomes.

## Data availability statement

The original contributions presented in the study are included in the article/Supplementary material, further inquiries can be directed to the corresponding author.

## Ethics statement

The studies involving human participants were reviewed and approved by the Beijing Hospital institutional review board. The patients/participants provided their written informed consent to participate in this study.

## Author contributions

DS and SW designed the study and wrote the manuscript. JW and JL helped with the study's design. JR, JC, and DW delineated the ROI and conducted the quality control of the image data. PQ had primary responsibility for the final content. All authors have read, critically revised, approved the final submitted manuscript, and agreed to be accountable for the content of the work.

## Supplementary material

Supplementary Figure S1 ICC test results. Features with an ICC value >0.9 were considered highly consistent and reliable and were thus selected for further research. These features were deemed to have minimal variability across imaging modalities and were considered to provide a stable representation of the underlying biological characteristics of the analyzed lesions. ICC Intraclass Correlation Coefficient.

Supplementary Figure S2 Comparison of Model Performance through the AUC Value. The ROC curves of model_RF and model_clinical on a testing cohort are presented together for a performance comparison through AUC value analysis. It can be observed from the figure that model_RF has a higher AUC value than model_clinical, indicating that model_RF outperforms model_clinical.
