# Supplementary figures and images for: Computed tomography angiography-based radiomics model for predicting carotid atherosclerotic plaque vulnerability
Source: Front Neurol. 2023 Jun 16;14:1151326. doi: 10.3389/fneur.2023.1151326 (PMC10312009; doi:10.3389/fneur.2023.1151326)

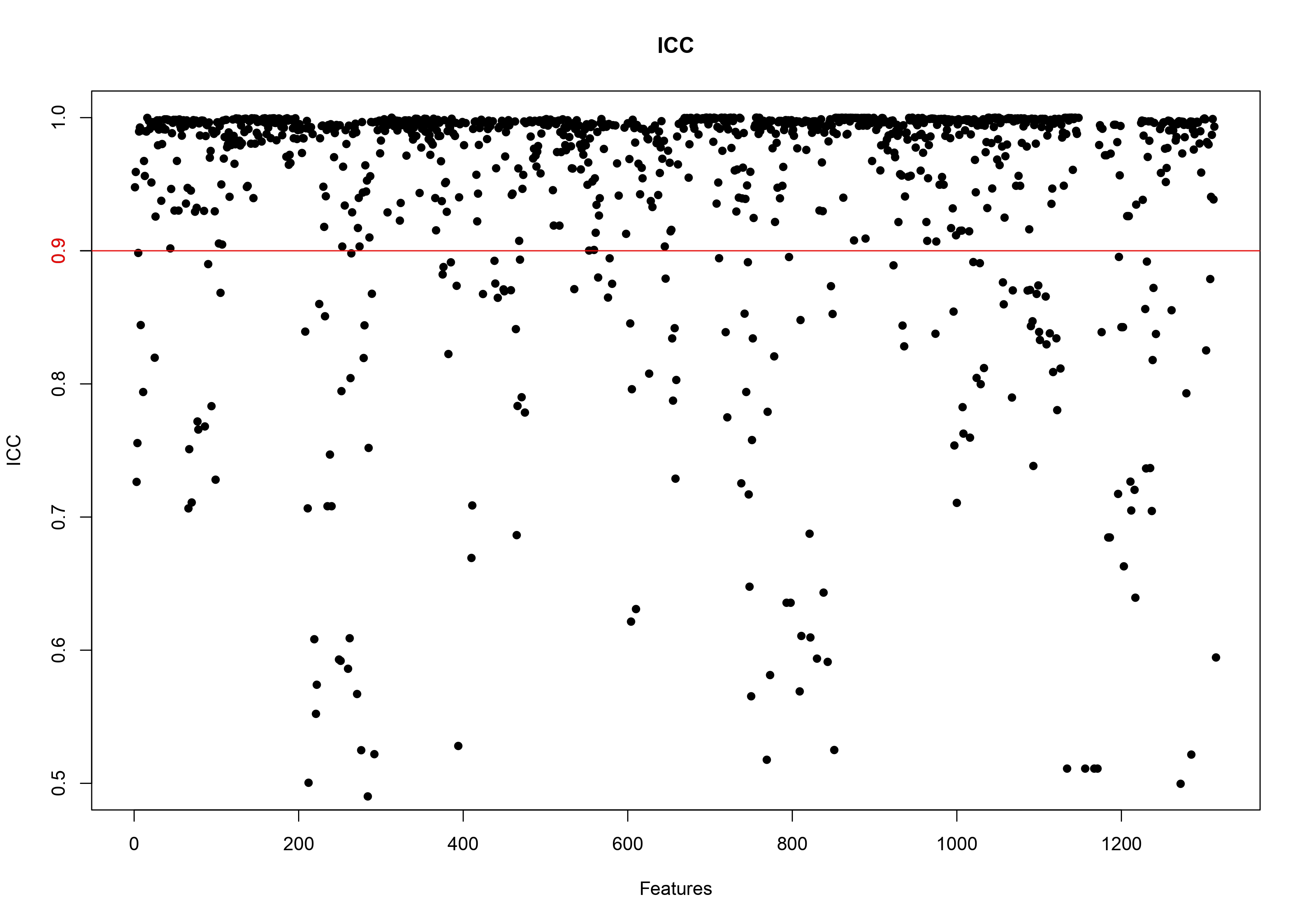

Supplement: Supplementary Figure S1 — ICC test results. Features with an ICC value >0.9 were considered highly consistent and reliable and were thus selected for further research. These features were deemed to have minimal variability across imaging modalities and were considered to provide a stable representation of the underlying biological characteristics of the analyzed lesions. ICC Intraclass Correlation Coefficient. [file Image_1.JPEG]

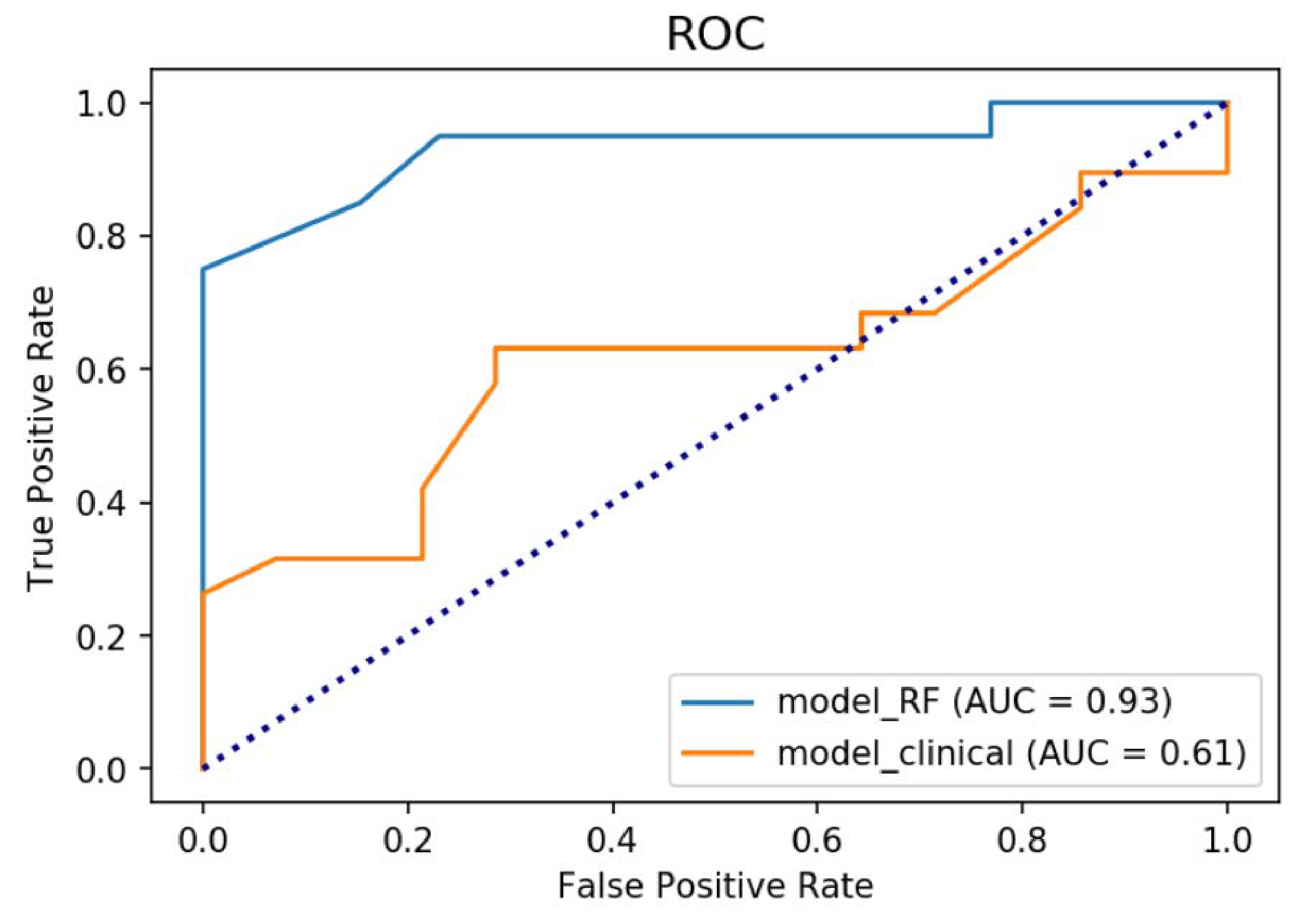

Supplement: Supplementary Figure S2 — Comparison of Model Performance through the AUC Value. The ROC curves of model_RF and model_clinical on a testing cohort are presented together for a performance comparison through AUC value analysis. It can be observed from the figure that model_RF has a higher AUC value than model_clinical, indicating that model_RF outperforms model_clinical. [file Image_2.JPEG]
